# MicroRNA-21 Is a Versatile Regulator and Potential Treatment Target in Central Nervous System Disorders

**DOI:** 10.3389/fnmol.2022.842288

**Published:** 2022-01-31

**Authors:** Xue Bai, Zhigang Bian

**Affiliations:** ^1^Department of Gerontology and Geriatrics, Shengjing Hospital of China Medical University, Shenyang, China; ^2^Department of Otolaryngology Head and Neck Surgery, Shengjing Hospital of China Medical University, Shenyang, China

**Keywords:** miR-21, central nervous system disorders, pathogenesis, biomarker, therapy

## Abstract

MicroRNAs (miRNAs) are a class of endogenous, non-coding, single-stranded RNAs with a length of approximately 22 nucleotides that are found in eukaryotes. miRNAs are involved in the regulation of cell differentiation, proliferation, invasion, apoptosis, and metabolism by regulating the expression of their target genes. Emerging studies have suggested that various miRNAs play key roles in the pathogenesis of central nervous system (CNS) disorders and may be viable therapeutic targets. In particular, miR-21 has prominently emerged as a focus of increasing research on the mechanisms of its involvement in CNS disorders. Herein, we reviewed recent studies on the critical roles of miR-21, including its dysregulated expression and target genes, in the regulation of pathophysiological processes of CNS disorders, with a special focus on apoptosis and inflammation. Collectively, miR-21 is a versatile regulator in the progression of CNS disorders and could be a promising biomarker and therapeutic target for these diseases. An in-depth understanding of the mechanisms by which miR-21 affects the pathogenesis of CNS disorders could pave the way for miR-21 to serve as a therapeutic target for these conditions.

## Introduction

Central nervous system (CNS) disorders are important causes of disability and death worldwide (De Feo et al., 2012). These neurological disorders are the results of intrinsic brain dysfunction or environmental interaction with brain (Spuch et al., 2012). CNS disorders affect 1.5 million people worldwide and responsible for 1% deaths (Soni et al., 2016). Clinically, the most common CNS disorders include cerebral vascular diseases, neurodegenerative diseases, CNS tumors, neuroautoimmune diseases, epilepsy, and CNS trauma. In any other disease, 11% of the burden of CNS disorders is reported and could increase to 14.7% by 2020 (Menken et al., 2000).

Various potential drugs have been discovered to treat CNS disorders. However, due to the presence of the blood-brain barrier (BBB), the therapeutic effects of these pharmaceuticals are still limited (Baird et al., 2021). BBB serves as an anatomical and biochemical dynamic barrier in the brain. It is composed of specific vascular endothelial cells and is tightly combined with pericytes, neurons and astrocytes (Tajes et al., 2014). Less than 1% of traditional drugs can cross this barrier (Stockwell et al., 2014). Therefore, the BBB can protect the brain from systemic circulating molecules and externally injected molecules, which poses a key challenge for drug delivery. Although there are many endogenous transporters in the nervous system, the BBB interacts with enzymes to make the treatment ineffective and restrict the entry of neurological drugs (Alam et al., 2010). Thus, a large number of patients with CNS disorders may suffer short-term or long-term disability (Spuch et al., 2012).

Emerging studies have indicated that microRNAs (miRNAs) are involved in the pathogenesis of CNS disorders and could serve as diagnostic biomarkers and therapeutic targets for these diseases (Gupta et al., 2017; Emamzadeh and Surguchov, 2018; Goh et al., 2019; Takousis et al., 2019; Si et al., 2020). In addition, because miRNAs have some desirable features for drug development, such as (1) one miRNA down-regulates hundreds of targets that bind to the 3′ UTR of target genes (Betel et al., 2010); (2) miRNA with a length of ∼22 nucleotides can be easy to design as miRNA drugs; (3) miRNA drugs can be delivered *in vivo* through several approved drug delivery systems used in humans (Rupaimoole and Slack, 2017), there are pharmaceutical companies have taken interest in miRNAs and used them as drug targets. Several miRNA drugs have entered human trials, such as RG-101, RG-125/AZD4076 and TagomiRs (Garzon et al., 2010).

miR-21 is one of the earliest discovered miRNAs in mammals (Kumarswamy et al., 2011). Numerous studies have shown that miR-21 plays important roles in the occurrence and development of various diseases (Chen et al., 2021; Olivieri et al., 2021; Surina et al., 2021). Apoptosis is a spontaneous programmed cell death that is regulated by multiple genes as a mechanism of adaptation to environmental changes (Abdulhussein et al., 2021). Abnormal changes in apoptosis can lead to some diseases, including CNS disorders (Kano et al., 2018; Xu et al., 2018; Zhao et al., 2020). It is reported miR-21 attenuated apoptosis-triggered by amyloid-β (Aβ) via modulating PDCD4/PI3K/AKT/GSK-3β pathway (Feng et al., 2018). A growing number of studies also suggest that inflammatory processes are involved in various neurological disorders (Rawji et al., 2016). miR-21-5p protects hippocampal neurons of epileptic rats via inhibiting STAT3 expression (Wang et al., 2017; Xu et al., 2019; Zhang et al., 2020) and is dysregulated in many diseases with abnormal apoptosis and inflammatory processes, especially in CNS disorders (Krichevsky and Gabriely, 2009; Liu et al., 2016; Sessa et al., 2019). The upregulated miR-21 increases cell survival, growth, and proliferation and decreases apoptosis and immune regulation (Li et al., 2016). In addition, miR-21 participates in other pathophysiological processes, such as autophagy, oxidative stress, and abnormal signal transmission (Fenoglio et al., 2011; Li et al., 2019a; Das et al., 2020). Substantial experimental evidence shows that differential expression of target genes of miR-21, caused by its dysregulation, increases the susceptibility to various CNS disorders. Moreover, miR-21 is suggested as a predictive and diagnostic biomarker for many CNS disorders, including Parkinson’s disease (PD), multiple sclerosis (MS), myasthenia gravis (MG), and epilepsy.

In this review, we summarize the latest research results on the expression levels, target genes, and mechanisms of action of miR-21 in different CNS disorders and elaborate on the important roles of miR-21 in different CNS disorders, as well as on its potential as a diagnostic biomarker and a new therapeutic target for these diseases.

## Production and Regulation of Expression of miR-21

Similar to other miRNAs, miR-21 is encoded by a specific gene, the MIR21 gene for humans, which is transcribed by RNA polymerase II in the nucleus to produce the initial pri-miR-21 transcript, which then forms a mature miR-21 through an orderly two-step modification. The initial transcript, pri-miR-21, is first cut into an approximately 72-nucleotide-long precursor miR-21 (pre-miR-21), with a stem-loop structure, by the Drosha enzyme from the RNA polymerase III family in cooperation with the DiGeorge syndrome critical region 8 (DGCR8) coenzyme (Davis et al., 2008). The pre-miR-21 is transferred from the nucleus to the cytoplasm by Exportin 5 and then modified by the Dicer enzyme from the RNA polymerase III family to produce a double-stranded miRNA: miRNA* duplex. miRNA*, which is difficult to unwind because of its high stability, is usually degraded, while miRNA, which is characterized by a low stability, is more likely to unwind and combine with the RNA-induced silencing complex (RISC) to form a functional miRISC complex, which binds to the 3′ UTR of the target mRNA to regulate mRNA expression (Borchert et al., 2006; Koo et al., 2015).

The miR-21 gene location is species specific. In humans, *MIR21* gene is located in the q23.1 region of chromosome 17, immediately downstream of the vacuole membrane protein-1 (VMP1) gene and resides within exon 10 of the *VMP1* gene (Ribas et al., 2012). Various transcription factors may be involved in the regulation of miR-21 expression, including activator protein 1 (AP-1), Ets/PU.1, CCAAT/enhancer-binding protein α (C/EBPα), nuclear factor I (NF-I), serum response factor (SRF), p53, and STAT3 (Misawa et al., 2010; Luo et al., 2014; McClure et al., 2014; Collins and Hess, 2016; Lee and Kim, 2020). However, different transcription factors have different regulatory signaling pathways for miR-21 expression. For example, the activation of AP-1 in 293FT cells activates the transcription of miR-21 with the assistance of PU.1, while the interaction between NFIB and C/EBPα inhibits the expression of miR-21, and there is a negative feedback regulatory relationship between miR-21 and NFIB (Fujita et al., 2008). In multiple myeloma, transcription factor STAT3 promotes the activation of an upstream enhancer of miR-21, and IL-6 induces the transcription of miR-21 (Krichevsky and Gabriely, 2009).

In addition to the regulation at the transcriptional level, the expression of miR-21 is regulated at the posttranscriptional level. Transforming growth factor-β (TGF-β) and bone morphogenetic protein 4 (BMP4) upregulate the expression of miR-21 by promoting the processing of pri-miR-21 transcripts by the Drosha enzyme (Ribas and Lupold, 2010). Moreover, the expression of miR-21 is also regulated by epigenetic mechanisms. For instance, hypermethylation of MIR21 in CD4 + T cells from patients with relapsing-remitting MS associates with lower miRNA-21 levels and concomitant up-regulation of its target genes (Ruhrmann et al., 2018).

## Roles of miR-21 in Central Nervous System Disorders

**FIGURE 1:**
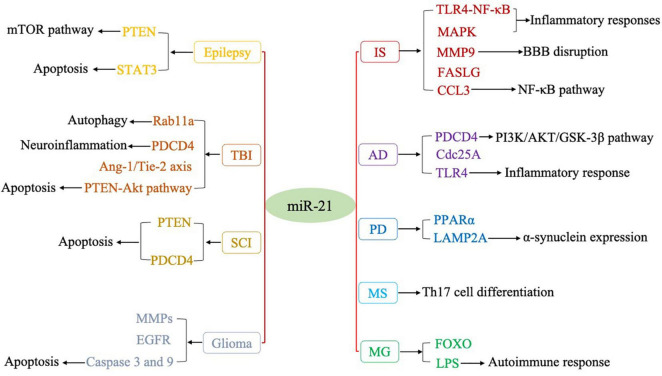
Regulatory targets of miR-21 in various CNS disorders.

Numerous studies have shown that miRNAs play important regulatory roles in the process of CNS disorders. There is convincing evidence that miR-21 (miR-21-5p) is closely associated with the regulation of almost all major CNS disorders, including ischemic stroke (IS), neurodegenerative diseases, CNS tumors, neuroautoimmune diseases, epilepsy, and CNS trauma. In terms of the mechanism, miR-21 may form a highly cooperative regulatory network with its target genes in various neuropathological processes (Figure 1). miR-21 mainly regulates the apoptosis and inflammatory processes in the nervous system and is also involved in astrocyte activation, glutamate toxicity, synaptic dysfunction, microglial burst activity, and remyelination (Table 1). Therefore, miR-21 ultimately affects the occurrence and development of CNS disorders.

**TABLE 1:** miR-21 functions in Neurological disorders.

Neurological disorders	Disease model	miRNA expression change	Targets	Biological meaning	References
Ischemic stroke	LPS induced macrophages	miR-21 up-regulated	TLR4-NF-κB pathway	Overexpression of miR-21 could reverse the pathological processes of atherosclerosis	Feng et al., 2014
Ischemic stroke	Rat model of cerebral I/R injury	miR-21 up-regulated	MAP2K3	Reduce the severity of cerebral edema, and abate the increase in BBB permeability disruption	Yao et al., 2018
Ischemic stroke	Rat model of ischemic stroke	miR-21 up-regulated	MMP9	Alleviate BBB disruption via a calcium-dependent mechanism.	Deng et al., 2013
Ischemic stroke	Rats subjected to embolic MCAo	miR-21-5p up-regulated	FASLG	Inhibit the hypoxia- and glucose deprivation-induced apoptotic cell death	Buller et al., 2010
Ischemic stroke	Rats with HIBD	miR-21-5p down-regulated	*CCL3*	Reduce the cerebral infarct volume and improve the neurobehavioral and memory abilities of rats	Liu et al., 2020
Alzheimer’s disease	APP/PS1 mice	miR-21 up-regulated	NA	Restorage synaptic dysfunction and regulate inflammatory responses	Cui et al., 2018
Alzheimer’s disease	APP695 (SH_Swe_) cells	miR-21-5p up-regulated	NA	Inhibit the release of microglial inflammatory factors	Fernandes et al., 2018
Alzheimer’s disease	Aβ_1–42_ in SH-SY5Y cells	miR-21 up-regulated	PDCD4	Repress cell apoptosis via PDCD4/PI3K/AKT/GSK-3β signaling pathway *in vitro*	Feng et al., 2018
Alzheimer’s disease	*A*β PPswe-PS1dE9 mice	miR-21 down-regulated	CDC25A	Regulate apoptosis and resist Aβ cytotoxicity	Chatterjee et al., 2016
Parkinson’s disease	SH-Y5Y cells	miR-21-5p up-regulated	PPARα	Improve the expression of PSD-95, BDNF, and GDNF	Fu et al., 2017
Parkinson’s disease	MPP^+^ induced MES cells	miR-21-5p up-regulated	NA	Inhibit the apoptosis and inflammatory responses	Mao and Ding, 2019
Parkinson’s disease	MPTP induced mice	miR-21-5p up-regulated	LAMP2A	Regulate α-synuclein and exhibit neuroprotective properties	Su et al., 2016
Amyotrophic lateral sclerosis	SOD1G93A	miR-21-5p down-regulated	NA	Modulate neuroinflammation and glial activation	Gomes et al., 2020
Multiple sclerosis	EAE mice	miR-21-5p up-regulated 5p	NA	Suppress neuroinflammation and attenuates clinical EAE development	Al-Ghezi et al., 2019
Multiple sclerosis	EAE mice	miR-21 up-regulated 5p	NA	Promote Th17 cell differentiation and mediate EAE symptoms	Murugaiyan et al., 2015
Myasthenia gravis	Murine macrophage cell	miR-21-5p up-regulated	FOXO	Inhibit of inflammatory response and corticosteroid therapy to MG	Fiorillo et al., 2020
Epilepsy	Kainic acid-induced rat	miR-21-5p up-regulated	PTEN	Modulate PTEN/mTOR pathway, decrease the number of neuronal deletions	Tang et al., 2018
Epilepsy	Epileptic rats	miR-21-5p up-regulated	STAT3	Inhibit STAT3 expression and reduce apoptosis and loss of hippocampal neurons	Zhang et al., 2020
Traumatic brain injury	HT-22 neurons	miR-21-5p up-regulated	RAB11A	Suppress RAB11A-mediated neuronal autophagy	Li et al., 2019a
Traumatic brain injury	Rat	miR-21-5p up-regulated	NA	Exerts the protective effect on BBB by activating the Ang-1/Tie-2 axis in BMVEC	Ge et al., 2015
Traumatic brain injury	Rat	miR-21 up-regulated	PTEN	Exert the function of reducing neuronal apoptosis through activating the PTEN-Akt signaling pathway	Han et al., 2014
Spinal Cord Injury	Rat	miR-21-5p down-regulated	PTEN/PDCD4	Enhance cell viability and suppress cell death via the miR-21/PTEN/PDCD4 signaling pathway.	Kang et al., 2019
Spinal Cord Injury	PC12 cells	miR-21-5p up-regulated	PTEN	Suppress the apoptosis of neuronal cells	Xu et al., 2019
Glioma	GBM cell	miR-21-5p up-regulated	RECK/TIMP3	Contribute to glioma malignancy by downregulating MMP inhibitors (RECK/TIMP3)	Gabriely et al., 2008
Glioma	GBM cell	miR-21-5p up-regulated	EGFR	Downregulation of miR-21 suppresses Glioblastoma Growth	Zhou et al., 2010a
Glioma	Human U87 glioma cell	miR-21-5p up-regulated	NA	Reduction of miR-21 by antisense oligonucleotides can activate caspase 9 and 3 pathway	Zhou et al., 2010b

### miR-21 and Ischemic Stroke

Ischemic stroke is one of the most important causes of neurological morbidity and mortality in the world (Maida et al., 2020). It is caused by an interruption of blood flow to the brain due to a clot and represents 87% of all stroke cases (Krishnamurthi et al., 2013). The plasma miR-21 levels are low in patients with early stage acute IS (AIS) and are negatively correlated with the National Institutes of Health Stroke Scale scores in the first day of AIS. However, the levels of miR-21 are upregulated in patients with AIS in the following days, and high levels may persist for quite a long time (Zhou and Zhang, 2014). Tsai et al. (2013) also showed that the serum miR-21 levels were significantly elevated in patients with stroke and atherosclerosis (Tsai et al., 2013). Among patients with aneurysmal subarachnoid hemorrhages, miR-21 also has relatively high levels in those with delayed cerebral ischemia (Bache et al., 2017).

Emerging evidence suggests that miR-21 is involved in the pathogenesis of IS via various mechanisms. Atherosclerosis is the main cause of the occurrence and progression of IS (Li et al., 2019b). Feng et al. (2014) demonstrated that miR-21 negatively regulated lipopolysaccharide (LPS)-induced lipid accumulation and inflammatory responses in macrophages via the Toll-like receptor 4 (TLR4)/NF-κB pathway. Overexpression of miR-21 could reverse the pathological processes of atherosclerosis, suggesting a promising therapeutic avenue for the prevention and treatment of atherosclerosis (Feng et al., 2014).

BBB disruption is involved in post-stroke edema and neurological deterioration in IS. Mitogen-activated protein kinases (MAPKs) are serine/threonine kinases that transmit signals from the cell membrane to the nucleus. MAPK signaling is related to the production of inflammatory cytokines and ischemia- and hemorrhage-caused neuronal death (Sun and Nan, 2016). In a rat model of cerebral I/R injury, upregulated miR-21 inhibited the MAPK signaling pathway by targeting MAP2K3 and attenuated neurological deficits, reduced the severity of cerebral edema, and abated the increase in BBB permeability disruption, thus potentially providing a new therapeutic target for the treatment of cerebral ischemia (Yao et al., 2018). Matrix metalloproteinase-9 (MMP9) also participates in BBB disruption and the formation of lesions in IS. MMP9 is considered to be critical in the pathogenesis of post-ischemic BBB disruption via the degradation of major components of the basement membrane that surrounds brain vessels. miR-21 was shown to be significantly upregulated in the rat hippocampus following cerebral ischemia, while an antagomir of miR-21 could decrease the MMP9 protein level after cerebral ischemia. Calcium signal stimulation may trigger the extracellular signal-regulated kinase signaling pathway by regulating miR-21, which leads to the upregulation of MMP9 after cerebral ischemia. Considering that miR-21 plays a key role in the occurrence and development of MMP9-mediated cerebral ischemia, anti-miR-21 may become a new effective intervention to prevent the formation of post-ischemic lesions (Deng et al., 2013).

Buller et al. (2010) showed that after stroke, the expression of miR-21 was upregulated in the neurons of the ischemic boundary zone compared with that in homologous contralateral neurons. In cultured cortical neurons, the overexpression of miR-21 significantly inhibited the hypoxia- and glucose deprivation-induced apoptotic cell death. Additionally, the overexpression of miR-21 decreased FASLG levels via specific binding to the 3′ UTR of FASLG. Thus, it has been suggested that overexpression of miR-21 protects against ischemic neuronal death and miR-21 may be an attractive therapeutic molecule for the treatment of IS (Buller et al., 2010).

Although the expression of miR-21 has frequently been reported to be elevated in many types of IS, the opposite result was also observed. Thus, Liu et al. (2020) detected that the expression of miR-21 was decreased in brain tissues of model rats with hypoxic–ischemic brain damage (HIBD). However, upregulation of miR-21 could play a neuroprotective role by reducing the cerebral infarct volume and improving the neurobehavioral and memory abilities of rats with HIBD via downregulation of *CCL3*, which has been identified to be a target gene of miR-21. In addition, the overexpression of miR-21 further disrupted the NF-κB signaling pathway by downregulating CCL3 expression (Liu et al., 2020).

Collectively, endogenous miR-21 expression levels are not consistent across different stages of IS, but most studies suggest that miR-21 expression continues to rise in the days after the occurrence of AIS. In addition, a consistent conclusion is that the upregulation of miR-21 expression can reduce the neurological deficit, decrease the size of cerebral infarction, and improve the prognosis of AIS. The neuroprotective effect of miR-21 upregulation is mainly due to its antiapoptotic, anti-inflammatory, and BBB protection mechanisms. However, it still needs to be further explored whether miR-21 could be used as a protective agent for IS and at what stage the miR-21 intervention may be most effective.

### miR-21 and Neurodegenerative Diseases

#### Alzheimer’s Disease

Alzheimer’s disease (AD) is a neurodegenerative disease with an insidious onset and a progressive course. AD is increasingly prevalent with advancing age, with a prevalence of 10–30% in those over 65, and incidence at least doubling every 10 years after 60 (Eratne et al., 2018). Extra-neuronal toxic amyloid oligomers and proteins, intraneuronal neurofibrillary tangles, region-specific diminished cerebral glucose metabolism, mitochondrial dysfunction, and inflammatory response are involved in the pathogenesis of AD (Oboudiyat et al., 2013). miR-21 is a key mediator of anti-inflammatory miRNAs and a negative regulator of TLR4 signaling. Microglial miR-21 can protect neurons from cell death under hypoxic conditions (Zhang et al., 2012; Sheedy, 2015; Fafián-Labora et al., 2017).

The expression of miR-21 was shown to significantly increase in mesenchymal stromal cells (MSCs) after hypoxic treatment. Exosomes from hypoxia-preconditioned MSCs could also effectively increase the level of miR-21 in the brain of C57BL/6 AD model mice. Moreover, upregulated miR-21 alleviated the cognitive deficits and pathological changes in APP/PS1 mice, suggesting that the intervention in inflammatory responses by regulating miR-21 expression may be a new approach for the treatment of AD (Cui et al., 2018).

Fernandes et al. (2018) demonstrated that the expression of miR-21 was upregulated in the Swedish mutant of APP695 (SH_Swe_) cells, and SH_Swe_-derived exosomal treatment also triggered sustained miR-21 expression by the human microglial clone 3 cell line. These results indicated an important role of miR-21 in neuron–microglia communication during AD pathogenesis and revealed the potential of miR-21 as a biomarker and therapeutic target, which may promote the development of personalized AD drugs (Fernandes et al., 2018).

Furthermore, it was found that miR-21 was significantly induced by Aβ_1–42_ *in vitro*, and miR-21 transfection inhibited Aβ_1–42_-induced SH-SY5Y cell apoptosis. Programmed cell death protein 4 (PDCD4) is an important tumor suppressor and has been reported to prevent AKT activation and to be involved in miR-21-repressed cell apoptosis in AD models (Feng et al., 2018). The phosphatidylinositol 3-kinase (PI3K)/AKT/GSK-3β pathway can release a survival signal to protect from multiple injuries (Ni et al., 2019). Upregulation of miR-21 was shown to increase PI3K, AKT, and GSK-3β activities, and a knockdown of PDCD4 could rescue their activities. Thus, it was suggested that miR-21 could exert protective effects in AD, which might be dependent on the PDCD4/PI3K/AKT/GSK-3β signaling pathway *in vitro* (Feng et al., 2018).

CDC25A plays an essential role in neuronal degeneration and death in AD and is elevated in neuronal cells by AD-relevant apoptotic stimuli. Aβ diminishes the levels of miR−21 in hippocampal and cortical neurons, while overexpression of miR-21 could sufficiently inhibit the CDC25A mRNA and protein induction by Aβ. Thus, targeting CDC25A by regulating miR-21 may be a useful strategy for providing neuroprotection in AD (Chatterjee et al., 2016).

In conclusion, the expression levels of miR-21 are not consistent in different AD models, but most experimental results support high expression levels of miR-21 in AD models. Overexpression of miR-21 plays a neuroprotective role, mainly via anti-inflammatory mechanisms. However, further *in vivo* studies are needed to determine whether miR-21 protects patients with AD from pathological damage.

#### Parkinson’s Disease

Parkinson’s disease (PD) affects 1–2 per 1,000 of the population at any time. PD prevalence is increasing with age and PD affects 1% of the population above 60 years (Tysnes and Storstein, 2017). PD is resulted from a pathophysiologic loss or degeneration of dopaminergic neurons in the substantia nigra of the midbrain, idiopathic PD is associated with risk factors including aging, pesticide exposure, family history, and environmental chemicals (Beitz, 2014). The levels of miR-21 are increased, and those of PPARα are decreased in patients with PD. Studies have shown that PPARα has a protective function in PD, and miR-21 is negatively correlated with PPARα in patients with PD. A luciferase activity assay further suggested that miR-21 could target the 3′ UTR of PPARα. Thus, downregulation of miR-21 may act as an epigenetic mediator for protecting neuronal cells by regulating PPARα expression (Fu et al., 2017).

miR-21 levels were markedly increased in MES cells following 1-methyl-4-phenylpyridinium (MPP^+^) treatment. Downregulation of miR-21 could reduce the MPP^+^-mediated cytotoxicity via inhibition of apoptosis and inflammatory responses in MES cells, which provides a novel approach to PD treatment (Mao and Ding, 2019).

Su et al. (2016) showed that the level of miR-21 was significantly increased in PD cells and mouse models. Lysosome-associated membrane protein 2 (LAMP2A) could increase chaperone-mediated autophagy, which is a main pathway of α-synuclein degradation (Xilouri et al., 2013). miR-21 upregulated the expression of α-synuclein by directly targeting the 3′ UTR of LAMP2A, whereas a miR-21 inhibitor effectively downregulated α-synuclein and exhibited neuroprotective properties (Su et al., 2016).

Accordingly, the expression level of miR-21 is increased in PD, and inhibition of miR-21 expression may protect PD neurons via various mechanisms. However, further evidence on the mechanism of miR-21 in PD is needed.

#### Amyotrophic Lateral Sclerosis

Amyotrophic lateral sclerosis (ALS) is a progressive neurodegenerative disease involving upper and lower motor neurons and is the most common form of adult motor neuron disease. The incidence of ALS is approximately 1–2.6 cases per 100,000 persons annually, whereas the prevalence is approximately 6 cases per 100,000 (Talbott et al., 2016). At present, the pathogenesis of ALS is unknown; however, copper and zinc superoxide dismutase gene mutations, excitatory amino acid toxicity, and neuroinflammatory responses may be involved in the pathogenesis of ALS (Kiernan et al., 2011; Brown and Al-Chalabi, 2017). Cunha et al. (2018) reported that the expression of miR-21 was elevated in SOD1^G93A^ mice. Vaz et al. (2019) found that the level of miR-21 was decreased upon the expression of the mutant SOD1 in N9 murine microglia. Recent studies have suggested that miR-21 is one of the most notorious inflammatory miRNAs (Quinn and O’Neill, 2011; Olivieri et al., 2013). miR-21 was shown to modulate the functional properties of astrocytes, and miR-21 expression may be age and region dependent (Rao et al., 2016). Gomes et al. (2020) observed decreased levels of miR-21 in cortical astrocytes and increased levels of miR-21 in spinal cord (SC)-derived astrocytes. This difference in miR-21 expression indicated regional heterogeneity of astrocytes, which may be helpful for understanding the role of astrocytes in the pathological mechanism of ALS (Gomes et al., 2020).

Thus, the expression levels of miR-21 are not consistent in different ALS cell models. miR-21 is involved in the pathogenesis of ALS mainly by regulating the function of immune cells, including microglia and astrocytes. Therefore, it is obvious that the expression levels of miR-21 are not consistent in different affected regions or immune states. However, few studies have explored the function of miR-21 in ALS, and thus, further studies on the mechanism of miR-21 in ALS are needed.

### miR-21 and Neuroautoimmune Diseases

#### Multiple Sclerosis

Multiple sclerosis (MS) is an autoimmune disease characterized by inflammatory demyelinating lesions of the white matter in the CNS (Oh et al., 2018), with a prevalence ranging from 2 per 100,000 in Japan to greater than 100 per 100,000 in Northern Europe and North America (Howard et al., 2016). Relapse-remitting multiple sclerosis (RRMS) is the most common MS phenotype, presenting as multiple lesions with remission and relapse over the course of the disease (Katz Sand, 2015). The pathogenesis of this disease has not yet been fully defined. In recent years, studies have proposed a multi-factor etiology theory of the combined effects of autoimmunity, viral infection, genetic predisposition, environmental factors, and individual susceptibility factors (Yamout and Alroughani, 2018). The levels of miR-21 are increased in cell-free cerebrospinal fluid (CSF) and the brain of patients with MS (Ma et al., 2014), and the elevated miR-21 levels are associated with active lesions in patients with MS (Muñoz-San Martín et al., 2019). In addition, miR-21 is downregulated in secondary progressive MS (SPMS) (Sanders et al., 2016) and upregulated in peripheral blood mononuclear cells in patients with relapsing-remitting MS (RRMS) (Fenoglio et al., 2011). Moreover, the levels of miR-21 in CD4^+^ T cells of patients with RRMS are lower than those in patients with SPMS.

miR-21 has been confirmed to be involved in cannabinoid-mediated suppression of inflammation (Sido et al., 2016). Al-Ghezi et al. (2019) suggested that miR-21 played a crucial role in experimental autoimmune encephalomyelitis (EAE) because downregulation of miR-21 attenuated EAE and miR-21 served as a neuroprotective mediator. Th17 cells are involved in various autoimmune diseases. Studies have demonstrated that the expression of miR-21 was elevated in Th17 cells and miR-21 promoted Th17 cell differentiation and mediated EAE symptoms (Xu et al., 2013; Murugaiyan et al., 2015). Meanwhile, treatment with an anti-miR-21 oligonucleotide reduced the clinical severity of EAE (Murugaiyan et al., 2015). Moreover, miR-21 was found to be an important regulator of Treg-triggered immune suppression (Zhou et al., 2015).

In summary, the levels of miR-21 were upregulated in most studies using different MS models. Thus, miR-21 might be a promising biomarker for MS diagnosis; in addition, miR-21 could be a biomarker to differentiate MS subtypes, for example, SPMS from RRMS. miR-21 may be involved in the pathogenesis of MS by promoting Th17 cell differentiation, and downregulation of miR-21 could serve as an indicator of therapeutic effects on EAE. However, further clinical studies are needed to verify the possibility of using miR-21 as a diagnostic biomarker, and *in vivo* experiments are needed to confirm its therapeutic value in MS.

#### Myasthenia Gravis

Myasthenia gravis (MG) is an autoimmune disease caused by neuromuscular junction transmission dysfunction, which is characterized by partial or systemic skeletal muscle weakness and fatigue (Gilhus, 2016). The global incidence rate of AChR positive MG ranges between 4 and 18 per million person-years (Bubuioc et al., 2021). MG is generally considered to be an autoimmune disease with an unknown etiology, which may be related to infection, drugs, and environmental factors (Gilhus and Verschuuren, 2015). The expression of miR-21-5p was shown to be specifically elevated in AChR Ab-seropositive MG, especially after a single bout of exercise (Punga et al., 2015; Westerberg et al., 2017). In addition, the level of miR-21-5p was increased in late-onset MG and decreased, in parallel with clinical improvement, after initiation of immunosuppressive therapy. Furthermore, the level of miR-21-5p is positively associated with the clinical MG composite score, being lower in patients with ocular MG than in those with generalized late-onset MG (Sabre et al., 2018). In addition, miR-21-5p is the most elevated miRNA in generalized AChR^+^ early onset MG, with lower levels observed after treatment with immunosuppressants and thymectomy (Punga and Punga, 2018; Sabre et al., 2020). Moreover, in a longitudinal randomized study, Molin et al. (2018) found that thymectomy-treated patients with MG had higher serum levels of miR-21-5p than those treated with prednisone-only. The levels of miR-21-5p displayed a negative correlation with the prednisone dose in prednisone-naïve patients in the prednisone group. Fiorillo et al. (2020) showed that the miR-21-5p levels were elevated in AChR^+^ patients with MG. A further study found a strong overall regulatory relationship between FOXO transcription factors and miR-21-5p. In a murine macrophage cell line, LPS was found to induce the expression of miR-21-5p via NF-κB-mediated inflammatory activation, strengthening the putative role of this miRNA in the MG autoimmune response (Fiorillo et al., 2020). In general, although its role has not been fully studied in the pathological mechanism of MG, miR-21-5p may be clinically applied as a promising diagnostic biomarker of MG.

#### miR-21 and Epilepsy

Epilepsy is an acute, recurrent, paroxysmal disorder of brain function caused by excessive discharge of neurons in the brain, with 1–2% of people around the world (Falco-Walter, 2020). Epilepsy is manifested as consciousness, movement, vegetative nerve, and mental disorders (Thijs et al., 2019). The expression of miR-21-5p was found to be higher in CSF and brain tissue of patients with temporal lobe epilepsy (TLE) and status epilepticus (SE) (Thijs et al., 2019). miR-21-5p levels showed good separation between SE and control samples. In addition, miR-21-5p levels were significantly higher in SE samples than in TLE samples and the control group, suggesting that miR-21-5p levels might discriminate between TLE and SE (Raoof et al., 2017).

The PTEN/mTOR signaling pathway is an important pathway in neurogenesis. In recent years, the mTOR pathway has been considered as a new target for the treatment of epilepsy (Switon et al., 2017). In a kainic acid-induced rat model of epileptogenesis, miR-21-5p was upregulated and PTEN was downregulated during acute, latent, and chronic stages of epileptogenesis compared with those in control rats. The PTEN/mTOR pathway is a target of miR-21-5p; knockdown of miR-21-5p decreased the number of neuronal deletions and improved the cognitive impairment caused by epilepsy, which suggested that the miR-21-5p/PTEN/mTOR axis might be a potential target for preventing and treating epileptic damage (Tang et al., 2018). Cortical dysplasia (CD) is a common cause of epilepsy in children and is characterized by deformed focal areas of the cerebral cortex (Iffland and Crino, 2017). As hsa-miR-21 was upregulated in CD and the mTOR signaling pathway was the most significantly associated pathway, Lee et al. (2014) suggested that the miR-21 was involved in the pathogenesis of CD, particularly in relation to the mTOR signaling pathway.

Apoptosis of hippocampal neurons is one of the main pathological changes in epilepsy (Prakash et al., 2019). STAT3 and the levels of cleaved caspase-3, BCL-2, and BAX are related to apoptosis of hippocampal neurons. Because miR-21-5p binds to STAT3, upregulation of miR-21-5p inhibits STAT3 expression and reduces apoptosis and loss of hippocampal neurons, thereby achieving protective effects on hippocampal neurons of epileptic rats (Zhang et al., 2020). miR-21 showed significant upregulation in acute and chronic stages in a rat model of mesial TLE (MTLE), while being downregulated in the latent stage. These different expression patterns may suggest different functions of miR-21 in MTLE pathogenesis (Peng et al., 2013).

In conclusion, there is consistent evidence that miR-21-5p levels are upregulated during epilepsy. Although reports are inconsistent, a targeted intervention to reduce miR-21-5p levels seems to affect the occurrence of epilepsy and may improve the prognosis. However, it is necessary to determine the optimal dose and time point of intervention. The behavior of patients and the pathology of epilepsy should also be systematically evaluated *in vivo*.

### miR-21 and Central Nervous System Trauma

#### Traumatic Brain Injury

Traumatic brain injury (TBI) is a common neurological disorder with high mortality, and the brain tissue damage is mainly caused by mechanical factors. After primary injury, various factors can lead to brain tissue edema and further distortion of brain tissue, followed by an increase of intracranial pressure and a decrease of the cerebral blood flow as secondary injury. Various pathophysiological mechanisms are involved in TBI, including inflammation, excitatory amino acid toxicity, oxidative stress, apoptosis, and other processes (Surgucheva et al., 2014; Niu et al., 2018). The levels of miR-21 are significantly increased in the serum of patients with TBI (Di Pietro et al., 2017) and are similar to those in the brains of TBI model mice (Lei et al., 2009; Ge et al., 2014).

Autophagy was reported to be persistently activated after TBI. Li et al. (2019a) suggested that autophagy was activated in HT-22 neurons after scratch injury; the expression of miR-21-5p was upregulated, and miR-21-5p could directly target the RAB11A 3′ UTR to reduce RAB11A expression and further suppress RAB11A-mediated neuronal autophagy. Neuroinflammation is one of the most important secondary events after initial TBI (Finnie, 2013). miR-21 is upregulated in microglia and dendritic cells and affects their differentiation after neuroinflammation (Sheedy, 2015; Harrison et al., 2016). In addition, miR-21 can directly target PDCD4 and participate in anti-inflammatory signaling by increasing the IL-10 level and decreasing that of IL-6 (Sheedy et al., 2010; van den Bosch et al., 2014), which suggests that miR-21 is involved in the initiation and maintenance of neuroinflammation. BBB damage is involved in secondary injury after TBI (Levin and Smith, 2013). Ge et al. (2015) demonstrated that the upregulation of miR-21 could exert a protective effect on the BBB by activating the ANG1/TIE2 axis in brain microvascular endothelial cells to prevent secondary BBB damage after TBI. Apoptosis of neuronal cells is an important pathological change in secondary brain injury, which is the key to functional recovery after TBI (Loane and Faden, 2010). miR-21 can reduce neuronal apoptosis by activating the PTEN/AKT signaling pathway, and more research is required to find other target genes of miR-21 in TBI (Han et al., 2014).

#### Spinal Cord Injury

SC injury (SCI) refers to SC damage caused by direct or indirect external factors and accompanied by various motor, sensory, and sphincter dysfunctions in the corresponding segments. In turn, the inflammatory response, immune damage, and cell apoptosis, secondary to SCI, exacerbate SC dysfunction (Gensel and Zhang, 2015). The expression of miR-21 was found to be decreased in SCI rats, while upregulation of miR-21 suppressed cell death and improved functional recovery of SCI rats via the miR-21/PTEN/PDCD4 signaling pathway (Kang et al., 2019). On the other hand, Ning et al. (2019) observed that the miR-21 levels were significantly increased in SCI rats, along with significant increases in the levels of inflammatory response-related factors, such as TNF-α and iNOS. In PC12 cells and MSCs from the treatment of SCI, the expression of miR-21 was upregulated, and it was suggested that the increased levels of miR-21 from differentiated PC12 cells and MSCs could suppress the apoptosis of neuronal cells by downregulating PTEN expression (Xu et al., 2019). In addition, miR-21 could exert an antiapoptotic effect by targeting PDCD4 to provide a protective role against neuronal apoptosis after SCI (Zhang et al., 2019). Other studies have found that miR-21 is upregulated after SCI, and this dysregulated expression can modulate the secretion, proliferation, and apoptosis of astrocytes to promote recovery after SCI (Bhalala et al., 2012; Liu et al., 2018). In general, the expression levels of miR-21 are not consistent in different SCI models, which may be related to the selected model and the detection time after SCI. Therefore, further *in vivo* studies on miR-21 as a diagnostic biomarker of SCI may be required. Nevertheless, most studies generally suggest that the upregulation of miR-21 after SCI can play a protective role on neurons via various mechanisms and may become a new therapeutic target.

#### miR-21 and Central Nervous System Tumors

Glioma is a common primary intracranial tumor (Abels et al., 2019). The miR-21 levels are significantly increased in the serum derived from patients with glioma (Ivo D’Urso et al., 2015), and miR-21 is also one of the most frequently overexpressed miRNAs in human glioma cell lines. miR-21 contributes to glioma malignancy by downregulating MMP inhibitors, thus promoting the invasiveness of cancer cells (Gabriely et al., 2008). Treatment of cells with antisense miR-21 was shown to decrease the expression of epidermal growth factor receptor (EGFR) and activate AKT, cyclin D, and BCL-2, which suggests that miR-21 may be a novel therapeutic target for malignant gliomas (Zhou et al., 2010a). In addition, downregulated miR-21 expression induced cell apoptosis and inhibited glioma cell proliferation. Moreover, a reduction in the miR-21 level led to the activation of caspase-3 and caspase-9, which may be mediated via multiple potential target genes (Zhou et al., 2010b). In conclusion, dysregulated expression of miR-21 is common in gliomas. miR-21 can affect the progression of gliomas via transcriptional and inflammatory mechanisms, and regulation of miR-21 expression may have a therapeutic effect on gliomas. However, the role of miR-21 in gliomas remains unclear and needs further study.

## Discussion and Perspectives

In this review, we focused on the roles of miR-21 in CNS disorders and its regulatory mechanisms in pathophysiological processes, especially in the context of apoptosis and neuroinflammatory responses. Our review illustrates the important potential of miR-21 as a diagnostic biomarker and therapeutic target in CNS disorders. Altered levels of miR-21 is a key factor in the pathogenesis of many CNS disorders, reflecting the extensive effects of miR-21 on the pathogenesis of these diseases. Most of the studies on miR-21 and CNS disorders have focused on the role of miR-21 in regulating crucial genes in apoptosis and neuroinflammatory responses, and the findings suggest that the level of miR-21 is correlated with these processes. However, it is important to note that the changes in the expression of miR-21 are not consistent in different stages of several diseases. The expression levels of miR-21 are elevated in most CNS disorders, including IS, AD, PD, MS, MG, and epilepsy. The study of the pathological mechanisms of various CNS disorders indicates that miR-21 can participate in the pathogenesis of CNS disorders via various mechanisms, including the regulation of cell apoptosis and neuroinflammatory processes. However, as the research progresses, there may be additional evidence of the involvement of miR-21 in the pathogenesis of CNS disorders.

The expression levels of miR-21 also vary in different tissues and body fluids, such as the brain, blood, and CSF. These changes are not surprising, considering that the BBB makes the biochemical conditions in the brain very different from those in the blood. Changes in tissue-specific miR-21 levels may be caused by events such as the damage to the BBB (Torres et al., 2011) and the feedback response of cells in different tissues to the disease state. The level of miR-21 in peripheral blood is more likely to be a potential biomarker for CNS disorders, while changes in the level of miR-21 in the brain are more likely to be conducive to targeted therapy. In addition, the expression levels of miR-21 are varied in different subtypes of the same disease (Table 2). For example, miR-21 is downregulated in patients with SPMS and upregulated in those with RRMS. miR-21 expression is increased in patients with AChR^+^ MG, but no significant change was observed in patients with other antibody-positive MG. Moreover, the expression of miR-21 may differ between patients and animal models with the same CNS disorders because of species differences or incomplete animal modeling methods. Therefore, the expression levels of miR-21 in human patients may better reflect the real clinical condition and may have broad prospects to serve as a diagnostic biomarker for CNS disorders. However, to apply miR-21 as a biomarker in clinical practice, large sample and multicenter studies are needed to further quantify its specificity and different types of changes in specific CNS disorders.

**TABLE 2:** Circulating miR-21 as potential biomarkers in CNS disorders.

CNS disorders	Samples	Expression	Function	References
AIS	Serum/plasm	Upregulated	Diagnosis of AIS, evaluation the severity of AIS patients in early stage of AIS.	Tsai et al., 2013; Zhou and Zhang, 2014
PD	Blood	Upregulated	Diagnosis of PD.	Fu et al., 2017
MS	CSF	Upregulated	Diagnosis of MS, prediction of active lesions in MS patients; diagnosis and distinguish between different subtypes of MS.	Fenoglio et al., 2011; Sanders et al., 2016; Muñoz-San Martín et al., 2019
SPMS	CD4^+^ T cells	Downregulated		
RRMS	PBMC	Upregulated		
MG	Serum	Upregulated	Diagnosis of AChR-Ab seropositive MG; distinguish early and late onset MG; distinguish ocular and generalized MG	Punga et al., 2015; Westerberg et al., 2017; Punga and Punga, 2018; Sabre et al., 2018, 2020
Epilepsy	CSF	Upregulated	Diagnosis of temporal lobe epilepsy and status epilepticus; discriminate temporal lobe epilepsy and status epilepticus.	Raoof et al., 2017; Thijs et al., 2019
TBI	Serum	Upregulated	Diagnosis of TBI	Di Pietro et al., 2017
Glioma	Serum	Upregulated	Diagnosis of glioma	Ivo D’Urso et al., 2015

In addition to being a promising diagnostic biomarker for various CNS disorders, miR-21 is one of the miRNAs that are most likely to become therapeutic targets. Based on previous studies on *in vivo* and *in vitro* models, upregulated expression of miR-21 appears to have neuroprotective effects in various CNS disorders. In IS, the overexpression of miR-21 could reverse the pathological processes of atherosclerosis (Feng et al., 2014), decrease the FASLG levels, and protect against ischemic neuronal death (Buller et al., 2010). In AD, upregulated miR-21 could alleviate cognitive deficits and pathological changes in APP/PS1 mice by inhibiting inflammatory responses (Cui et al., 2018). In epilepsy, the upregulation of miR-21-5p inhibited STAT3 expression and reduced apoptosis and the loss of hippocampal neurons, thereby achieving protective effects on hippocampal neurons of epileptic rats (Zhang et al., 2020).

Collectively, miR-21 can improve the prognosis of CNS disorders by inhibiting cell apoptosis and inflammatory processes. However, we still face a number of practical challenges before miR-21 can be used in clinical practice. First, miR-21 may be involved in the pathogenesis of CNS disorders via various mechanisms and has regulatory relationships with pathogenic genes in various disease models. Therefore, it is necessary to further clarify the pathological mechanisms of miR-21 in different CNS disorders for developing miR-21 as an accurate therapeutic target. Second, because of the existence of the BBB, the effective expression of miR-21 in the affected areas of patients with CNS disorders is another problem that needs to be resolved. The development of viral vectors (adenovirus) and non-viral vectors (liposomes and nanocarriers) could be helpful for the early application of miR-21 in human clinical trials. Lastly, although miR-21 has shown neuroprotective effects in various CNS disorders in animal and cell models, dose evaluation in humans is still lacking. Therefore, there is still a long way to go for miR-21 to be applied as a therapeutic agent for clinical treatment of CNS disorders.

In conclusion, miR-21 is involved in the progression of CNS disorders via various mechanisms, among which apoptosis and inflammatory processes play important roles, and miR-21 may potentially be used as a biomarker and therapeutic target for CNS disorders. However, there are still many problems to be solved before the clinical application of miR-21. Further understanding of the mechanisms by which miR-21 intervenes in apoptosis and regulates inflammatory processes in different CNS disorders will be helpful for the use of this miRNA as a therapeutic target in CNS disorders.

## Author Contributions

XB and ZB drafted and revised the manuscript. ZB drafted and modified the figures. Both authors approved the final version of the manuscript.

## Conflict of Interest

The authors declare that the research was conducted in the absence of any commercial or financial relationships that could be construed as a potential conflict of interest.

## Publisher’s Note

All claims expressed in this article are solely those of the authors and do not necessarily represent those of their affiliated organizations, or those of the publisher, the editors and the reviewers. Any product that may be evaluated in this article, or claim that may be made by its manufacturer, is not guaranteed or endorsed by the publisher.
